# Efficient prioritization of CRISPR screen hits by accounting for targeting efficiency of guide RNA

**DOI:** 10.1186/s12915-023-01536-y

**Published:** 2023-02-24

**Authors:** Byung-Sun Park, Heeju Jeon, Sung-Gil Chi, Tackhoon Kim

**Affiliations:** 1 Medicinal Materials Research Center, Korea Institute of Science and Technology, 5 Hwarangro-14-Gil, SeongbukGu, Seoul, 02792 Republic of Korea; 2 Department of Biological Sciences, Korea University, 145 AnamRo, SeongbukGu, Seoul, 02841 Republic of Korea; 3 Division of Bio-Medical Science and Technology, Korea University of Science and Technology, 217 GajeongRo YuseongGu, Daejeon, 34113 Republic of Korea

**Keywords:** CRISPR-Cas, Functional genomics, CRISPR screens, Melanoma, Drug resistance

## Abstract

**Background:**

CRISPR-based screens are revolutionizing drug discovery as tools to identify genes whose ablation induces a phenotype of interest. For instance, CRISPR-Cas9 screening has been successfully used to identify novel therapeutic targets in cancer where disruption of genes leads to decreased viability of malignant cells. However, low-activity guide RNAs may give rise to variable changes in phenotype, preventing easy identification of hits and leading to false negative results. Therefore, correcting the effects of bias due to differences in guide RNA efficiency in CRISPR screening data can improve the efficiency of prioritizing hits for further validation. Here, we developed an approach to identify hits from negative CRISPR screens by correcting the fold changes (FC) in gRNA frequency by the actual, observed frequency of indel mutations generated by gRNA.

**Results:**

Each gRNA was coupled with the “reporter sequence” that can be targeted by the same gRNA so that the frequency of mutations in the reporter sequence can be used as a proxy for the endogenous target gene. The measured gRNA activity was used to correct the FC. We identified indel generation efficiency as the dominant factor contributing significant bias to screening results, and our method significantly removed such bias and was better at identifying essential genes when compared to conventional fold change analysis. We successfully applied our gRNA activity data to previously published gRNA screening data, and identified novel genes whose ablation could synergize with vemurafenib in the A375 melanoma cell line. Our method identified nicotinamide N-methyltransferase, lactate dehydrogenase B, and polypyrimidine tract-binding protein 1 as synergistic targets whose ablation sensitized A375 cells to vemurafenib.

**Conclusions:**

We identified the variations in target cleavage efficiency, even in optimized sgRNA libraries, that pose a strong bias in phenotype and developed an analysis method that corrects phenotype score by the measured differences in the targeting efficiency among sgRNAs. Collectively, we expect that our new analysis method will more accurately identify genes that confer the phenotype of interest.

**Supplementary Information:**

The online version contains supplementary material available at 10.1186/s12915-023-01536-y.

## Background

CRISPR-Cas9 screens [1–4] have been widely adopted to discover novel therapeutic targets, whose disruption leads to a favorable phenotype in relevant disease models. For instance, crucial factors that modulate cancer immunotherapy [5], as well as other diseases, including ferroptosis-associated lipid peroxidation [6] and hemoglobinopathies [7] have been discovered. CRISPR-Cas9 screens are widely used as viability screens for cancer research to search for genes whose ablation can decrease cancer cell fitness.

CRISPR-Cas9 screens, especially dropout screens that identify the sgRNAs that are depleted in the population, typically yield tens to hundreds of candidate “hits.” Therefore, efficient prioritization of the hits among those candidates for further validation can enhance efficiency in finding genes whose ablation reduces cellular fitness. However, low-activity sgRNAs inevitably add bias to the screening results, leading to false negative hits. Different sgRNAs targeting the same gene often result in varying changes in the phenotype despite the assumption underlying CRISPR-Cas9 screens that the change in phenotype by sgRNA expression is purely a consequence of the ablation of the sgRNA target gene. Several attributes of sgRNAs have been proposed to contribute to the noise and bias in CRISPR screens, such as (i) sgRNAs can induce off-target effects by targeting other unintended gene sites [8–11]; (ii) the copy number of targeted genes varies in cells, especially in the cancer cells [12, 13]; and (iii) efficiencies of insertions and deletions (indels) differ among gRNAs [14]. Therefore, one of the most crucial tasks in the discovery of target genes using CRISPR screens is minimizing the noise and bias in CRISPR-Cas9 screens to maximize the likelihood of finding true hits within the candidate hits.

Several approaches have been developed to refine CRISPR screening results. Doench and colleagues have used an increasing number of sgRNAs targeting the same gene [15]. Elling and colleagues and other groups used a unique molecular identifier (UMI) to lineage trace single cell in CRISPR screens to remove outlier cells with aberrant behaviors [16–18]. Also, Garnett and colleagues and others made extensive characterization of the standard sgRNAs widely used to generate minimized sgRNA library consisting of the most effective guides [13, 19]. Many groups have developed rules that govern the on-target and off-target activities of sgRNAs to successfully predict them [15]. However, considering that sgRNA sequence design is restrained by the presence of PAM and the sequence of the target gene, it is practically impossible to expect that thousands of sgRNAs used in CRISPR screens are all perfectly optimized. In addition, sgRNA optimization rules must be established for each Cas nuclease, including the less characterized Cas nucleases [20] and engineered SpCas9 nucleases [21–23], limiting generalizability.

**Fig. 1:**
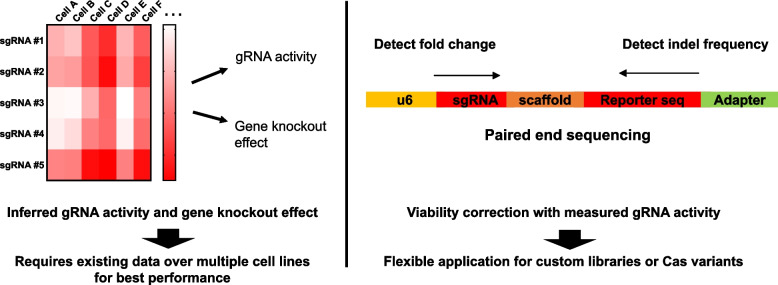
Approaches for correcting sgRNA activity in CRISPR screens. The conventional method for adjusting guide RNA activities based on the pre-existing CRISPR screening database (left). The new method enables efficient correction of CRISPR screen results only with the detection of fold change and indel frequencies of gRNAs from the specific libraries (right)

Parts and colleagues and Tsherniak and colleagues developed methods to infer sgRNA activity from published CRISPR screen data to correct the screen results [24, 25]. The inference requires an established set of CRISPR screen results across multiple cell lines for best performance. Therefore, while this approach may be useful for correcting CRISPR screen data with established sgRNA library with records of well-validated results, such data are again limited for CRISPR screens with custom sgRNA libraries or with less characterized Cas nucleases. We reasoned that the above limitations in optimization and correction of CRISPR screens can be overcome if the frequency and DNA cleavage efficiency of each sgRNA can be measured simultaneously (Fig. 1). This approach enables efficient correction of CRISPR screen results without existing knowledge in sgRNA activity or requiring multiple replicates across multiple cell lines because each replicate contains both the changes in viability and frequency of indel mutations in cells carrying a specific sgRNA.

By taking a library architecture that enables such a task [26], we present a novel analysis method for adjusting changes in viability with the actual, observed differences in indel frequencies. By performing a “tiling array” screen that involves the use of every possible sgRNA that targets seven different genes, we confirmed that the variation in sgRNA activity is by far the dominant factor contributing to bias in the results. We further developed and validated an analysis method to correct the viability score based on differences in sgRNA activity. We applied our new analysis method to previously published CRISPR screens to improve the receiver operating characteristic area under the curve (ROC-AUC) value for the true positive discovery of essential genes exceeding 0.983. We expect our method to provide a framework for removing bias in CRISPR screens for more efficient prioritization of screen hits for further validation where pre-existing evidences of sgRNA activities are limiting.

## Results

### Indel generation efficiency of sgRNA is the dominant factor influencing the changes in phenotype

**Fig. 2:**
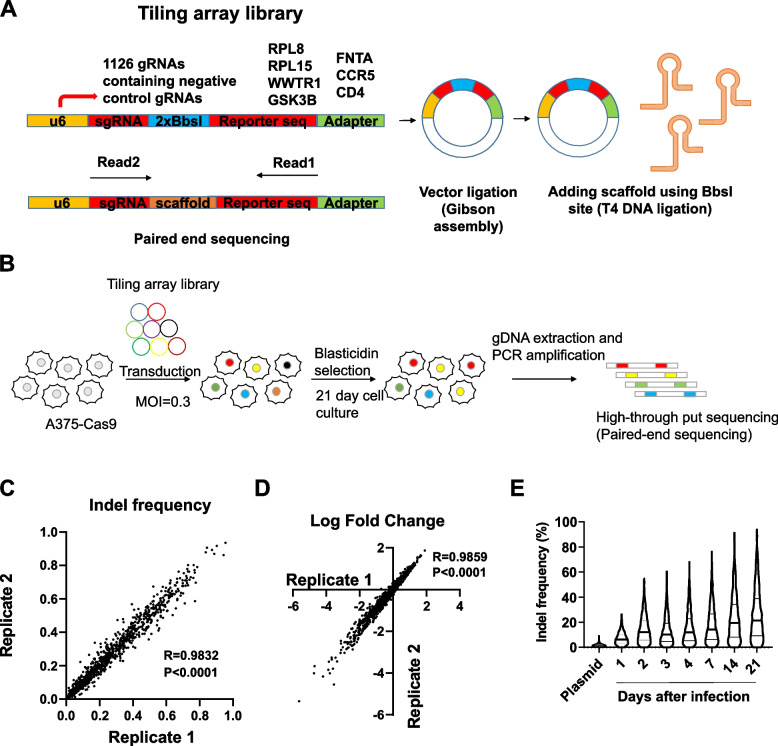
Schematic design and data quality control of tiling array library CRISPR screening. **A** Schematic diagram of the library construction procedure and paired-end sequencing for simultaneous detection of guide RNA and the reporter sequence. **B** Tiling array library CRISPR screening workflow. **C** Correlation of indel frequencies between two replicates of day 21 samples. **D** Correlation of log fold changes between two replicates of day 21 samples. *R* indicates Pearson correlation coefficient* r* and *p* value is calculated by two-tailed test. **E** Indel frequencies at indicated timepoints from the tiling array library. The black line indicates the median indel frequency

We first identified the contribution of each sgRNA attribute to the bias in the CRISPR screen results. To this end, we generated a tiling array library that contained all possible sgRNAs targeting seven selected genes. The selected genes included two essential genes (RPL8 and RPL15), two dispensable genes (CCR5 and CD4), and three genes that were expected to give an intermediate phenotype (FNTA, WWTR1, GSK3B). As previously described [26], each sgRNA within the library was paired with a sequence (hereafter called the “reporter sequence”) that can be targeted by the same sgRNA (Fig. 2A). If the sgRNA is active and can cleave the endogenous target gene, the sgRNA will similarly generate an indel mutation in the reporter sequence. The library construction was done with fold coverage of at least 500 to preserve even representation of sgRNAs. Also, PCR amplification of the oligonucleotide pool was optimized to minimize the decoupling of sgRNA and reporter sequence (see the “Methods” section) [27]. We confirmed that > 70% of reporter sequences were correctly paired with their corresponding sgRNAs using deep sequencing (Additional file 1: Fig. S1A). We delivered this library into an A375 melanoma cell line stably expressing Cas9. The activity of Cas9 was confirmed in at least 87% of cells using flow cytometry (Additional file 1: Fig. S1B). The indel mutations in the reporter sequence and endogenous target were well correlated (Additional file 1: Fig. S1C). A375-Cas9 cells were infected with the lentiviral library and were collected at multiple time points for 3 weeks for genomic DNA extraction and deep sequencing (Fig. 2B).

Paired-end sequencing of the PCR amplicon derived from the lentiviral genome could simultaneously identify sgRNA and the presence of indel mutations in the reporter sequence (Fig. 2A). NGS analysis revealed that most sgRNAs are well represented with 89% (962/1086) and 97% (1045/1086) of sgRNA frequencies within library within twofold and fivefold difference from median frequency (0.93 reads per thousand), respectively (Additional file 1: Fig. S1D). 2.6% (28/1086) of sgRNAs with reads per thousand of less than 0.1 were excluded from analysis as these sgRNAs showed large variation in sgRNA frequency and indel frequency between biological replicates, indicating genetic drift (Additional file 1: Fig. S1E, F). Fold changes (FC) in the frequency of each sgRNA and the fraction of reporter sequences that had been mutated had a very good correlation (Pearson’s *r* = 0.98, *p* < 0.0001) between biological replicates, highlighting the reproducibility of the screening approaches (Fig. 2C, D). FC and mutation frequency of the reporter sequence were correlated with viability scores in previously published screen results (Additional file 1: Fig. S1G) and the predicted on-target cleavage efficiency score (Additional file 1: Fig. S1H), respectively. We observed a gradual increase in indel frequency over time (Fig. 2E), indicating that the saturation of the indel mutation was minimal during the experiment. The indel frequencies varied between 0 and 94%, reflecting that the library consisted of an unoptimized set of sgRNAs.

**Fig. 3:**
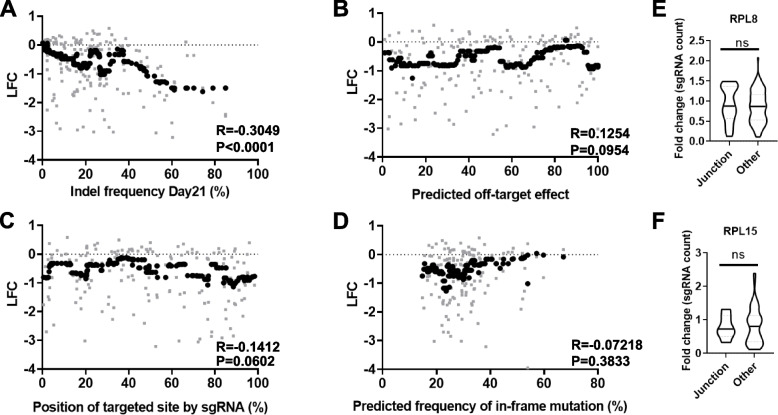
Indel frequency influences log fold change (LFC) viability scores. **A**–**D** Correlation between moving median of LFC in the frequency of sgRNAs targeting RPL8 or RPL15 and **A** indel frequency (day 21), **B** predicted off-target rank percentage, **C** percentage of gRNA-targeting cut sites within target gene coding sequences, and **D** percentage of predicted in-frame mutations. Gray dots indicate values for each individual sgRNA while black bold dots indicate the moving median of the 20 nearest neighbors. *R* indicates Pearson correlation coefficient *r* and *p* value is calculated by the two-tailed test. **E**, **F** Comparison of sgRNA fold changes between sgRNAs targeting near exon–intron junction (Junction) and those targeting within exon (Other)

We first assessed the effect of several attributes of the sgRNA, including indel frequency, off-target effect, and positions of the sgRNA target sequences within the gene sequence, on the FC values for each sgRNA. The FC showed a negative correlation with the indel frequency of the reporter sequence (Pearson’s *r* =  − 0.30, *p* < 0.0001, Fig. 3A, Additional file 1: Fig. S2). In contrast, the predicted number of off-targets, position of targeted sites within the coding sequence, predicted frequency of in-frame mutation, and sgRNA specificity scores had little, if any, influence on FC values (Pearson’s *r* =  − 0.14–0.13, *p* > 0.05, Fig. 3B–D). sgRNAs whose target sites are spanning the exon–intron junctions [28] did not show any significant differences in fold change compared to those targeting within an exon (Fig. 3E, F). These results suggest that indel frequencies of the gRNAs are by far the most dominant factor in determining the FC of sgRNAs. This prompted us to develop a new analysis method to correct the bias.

### Mutations in the reporter sequence are coupled with those in the endogenous target gene in the same cell

**Fig. 4:**
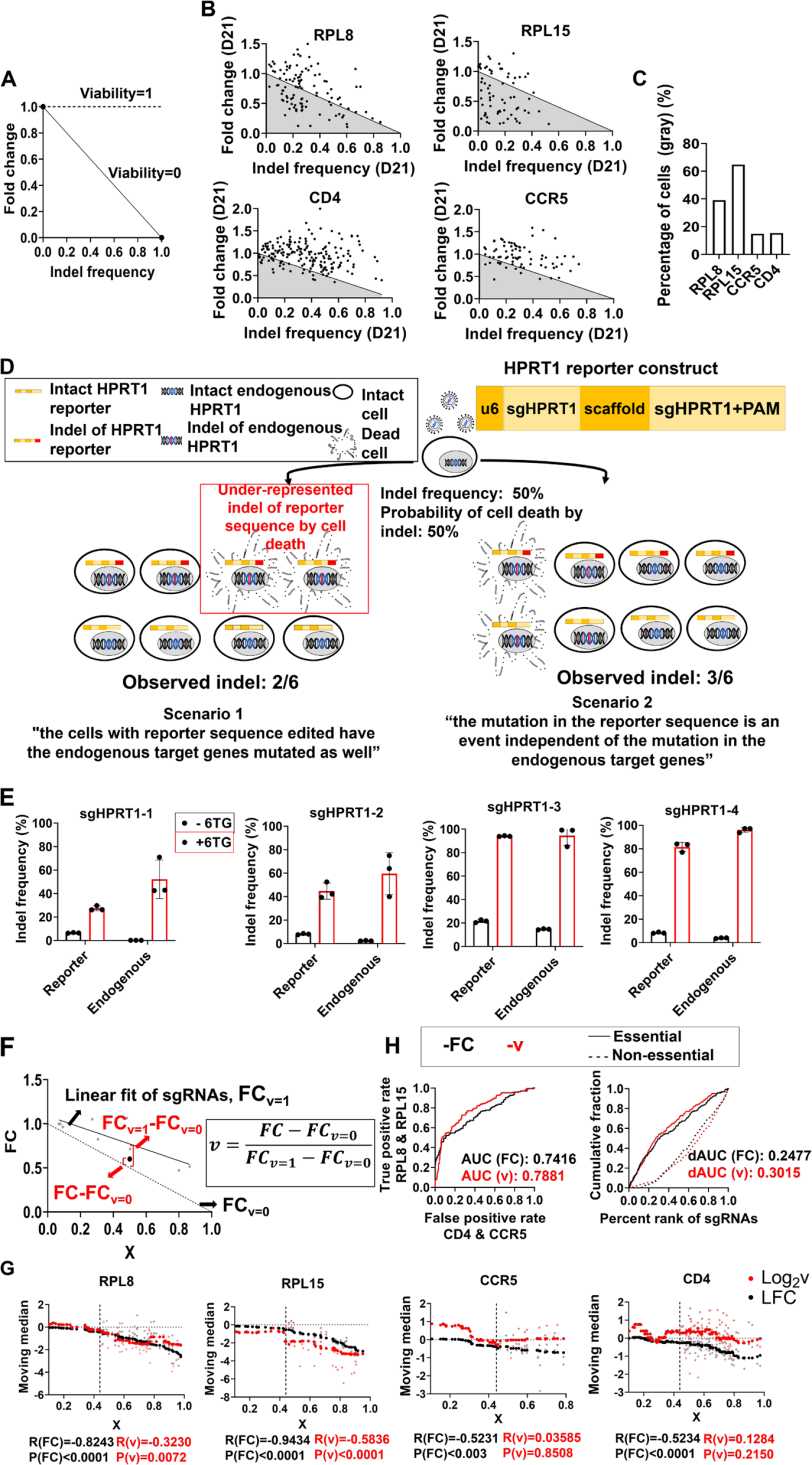
New analysis method with *v* metric reduces bias generated from the conventional method. **A** Theoretical minimum fold change as a function of indel frequency (viability = 0). **B** Scatter plots for indel frequency and fold change at day 21 for sgRNAs targeting indicated genes. The black line indicates the theoretical minimum FC at viability = 0. Gray shade indicates the area below theoretical minimum FC. **C** Quantification of cells at expected viability below 0 from **B** (gray). **D** Two theoretical scenarios showing the relationship between the reporter sequence and endogenous sgRNA target gene. **E** Quantification of indel frequencies of the reporter sequence and endogenous HPRT1 gene using four different gRNAs targeting HPRT1 in the presence and absence of 6-TG. **F** Linear regression of indel frequency and fold change at day 21 of negative control gRNAs (solid line). The dotted line is an expected line at viability = 0. Gray dots indicate individual points of negative control sgRNAs and the black bold dot indicates a hypothetical point. **G** The moving median of 4 genes according to *X* analyzed by LFC or Log_2_***v***. All data are presented as mean ± s.d. *n* = 3 biological replicates. Pearson correlation coefficient *r* is represented by *R* with *X* > 0.44 cutoff and the *p* value is calculated by a two-tailed test. **H** The receiver operating characteristic (ROC) curve of the two analysis methods. The true positive rate is decided by RPL8 and RPL15 and plotted against the false positive rate, decided by CCR5 and CD4 (left). The area under the curve analysis for essential (solid line) and non-essential (dotted line) genes (right)

We recognized that there are sgRNAs with a very low FC and indel frequency. Cells expressing inactive or low-activity sgRNA should have viability comparable to those expressing non-targeting sgRNA. Therefore, the theoretical minimum FC value for each sgRNA (assuming zero viability for cells that have indel mutation in the target sgRNA gene) is *1-indel* *frequency*. However, the FC values of sgRNAs, especially those targeting essential genes (RPL8 and RPL15), fell far below the theoretical minimum, leading to an unacceptable conclusion that the viability of cells with target gene cleaved is below zero (Fig. 4A–C, Additional file 1: Fig. S3A-B). Hence, we hypothesized that the indel frequency is particularly underestimated for sgRNAs targeting genes whose disruption causes significant decreases in viability. To this end, we hypothesized that the mutation of the reporter sequence is coupled with that of endogenous target genes. Therefore, cells expressing sgRNA against essential genes with the reporter sequence and sgRNA target gene mutated will quickly be eliminated from the population, leading to the underrepresentation of indel mutation in the reporter sequence (Fig. 4D).

To test our hypothesis, we performed a 6-thioguanine (6-TG) selection assay [15]. 6-TG is metabolized by HPRT1 to thioguanosine monophosphate, which blocks purine biosynthesis and ultimately causes DNA damage and toxicity [29]. Therefore, ablation of HPRT1 prevents cell death after 6-TG treatment. If the mutation of the reporter sequence is independent of that of endogenous genes, the frequency of the mutated reporter sequence will be the same regardless of the 6-TG selection. Alternatively, if the mutation is a reporter sequence coupled with that of endogenous genes, the cells that survived 6-TG selection and those with endogenous HPRT1 mutations will have the reporter sequence mutations as well. We constructed vectors containing an HPRT1-targeting sgRNA coupled with a reporter sequence that could be targeted by the same sgRNA. Surprisingly, we found that up to 94% of cells that survived the 6-TG treatment had a mutated reporter sequence, suggesting that the mutation of the reporter sequence and that of endogenous sgRNA target genes were coupled (Fig. 4E). This is remarkable as the reporter sequence is located at random location independent of endogenous sgRNA target in HPRT1 gene. Any changes in the frequency of sgRNA, which is a consequence of the change in viability upon mutation of the endogenous target gene, will also change the indel frequency by the same magnitude (Fig. 4D). Therefore, the observed indel is underrepresented by FC-1 when compared with the actual indel frequency. This led us to define an adjusted, actual indel frequency denoted as “*X*,” as a function of the FC and observed indel. The equation for “*X*” is given below:

$$X=1-\mathrm{FC}+\mathrm{FC}\times \mathrm{indel}$$

### Establishing a model for inferring viability upon gene perturbation from the changes in sgRNA frequency

We then established a model scenario that can be used to infer the actual viability*** v*** upon ablation of the sgRNA target gene from the changes in sgRNA frequency within the library. Any changes in the frequency of each sgRNA were a consequence of the change in viability*** v*** − 1 in the *X* fraction of the total population carrying the given sgRNA. Therefore, the FC, the observed fold change in sgRNA frequency in CRISPR screens, can be described as a function of*** v*** and *X* as follows:

$$\mathrm{FC}=\left({\varvec{v}}-1\right)\times X+1$$

In this model, FC is equal to 1 and 1 − *X* when*** v*** is 1 and 0, respectively. The deviation of FC from 1 − *X* is proportional to ***v***. Therefore,*** v*** can be calculated as

$${\varvec{v}}=\frac{\mathrm{FC}-\left(1-X\right)}{X}$$

We first tested whether using the new viability metric*** v*** as the actual viability could attenuate bias. Contrary to our expectation,*** v*** still showed a clear negative correlation with *X*, even for genes whose ablations are not expected to affect the viability (e.g., CD4 and CCR5) (Additional file 1: Fig. S3C, D). In search of the cause of this correlation, we found that even*** v*** values for the negative control sgRNAs showed a negative correlation with *X*. This correlation may be a consequence of the DNA damage response and subsequent proliferation arrest upon indel mutations [30, 31]. The sgRNAs with higher cleavage efficiencies may also have higher off-target cleavage frequencies.

To adjust the changes in FC by simple negative correlation with *X*, we used the linear regression of FC and *X* values of the negative control sgRNAs as the hypothetical FC values when*** v*** = 1. With FC_***v***=1_ as the expected FC when*** v*** = 1 inferred from the negative control sgRNAs (Fig. 4F), we newly defined the viability*** v*** for any given sgRNA as follows:

$${\varvec{v}}=\frac{\mathrm{FC}-{\mathrm{FC}}_{{\varvec{v}}=0}}{{\mathrm{FC}}_{{\varvec{v}}=1}-{\mathrm{FC}}_{{\varvec{v}}=0}}=\frac{\mathrm{FC}-\left(1-X\right)}{{\mathrm{FC}}_{v=1}-\left(1-X\right)}$$

### The new analysis method reduces bias in the phenotype score of sgRNAs in screens with custom sgRNA library

We examined whether the use of our new viability metric*** v*** can attenuate the bias in the viability score by indel frequency. Actual viability*** v*** values had a much weaker correlation with* X* values than with FC within the range of moderate to high *X* values (*X* > 0.44) (Pearson’s *r*_FC_ =  − 0.94 to − 0.52, *r*_***v***_ =  − 0.32–0.13; Fig. 4G). Notably, the*** v*** values tended to increase with decreasing *X* values for the *X* values less than 0.44. This may be due to noise in the FC values generated by the off-target effect, which can be amplified with very low *X* values (see the “Discussion” section). We also tested whether sgRNAs targeting the essential genes RPL8 and RPL15 were more efficiently identified with*** v*** than with FC in our tiling array screens. Remarkably, the*** v*** score more efficiently identified RPL8 and RPL15 as essential genes with an increase in the ROC-AUC value of 6.27% and an increase in delta AUC value, which quantifies the ability in distinguishing essential and non-essential genes within library [32] by 21.7% (Fig. 4H). The AUC values were lower than those previously reported in genomewide screens, largely because the library mainly consisted of unoptimized sgRNAs.

**Fig. 5:**
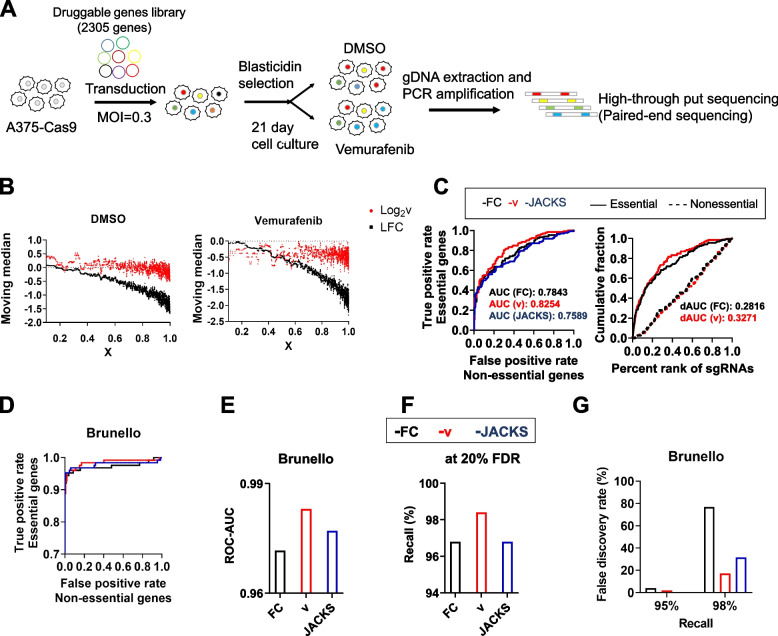
sgRNA activity-corrected viability metric *v* more efficiently identifies essential genes. **A** Druggable gene library CRISPR-Cas9 screening workflow. **B** The moving median of all sgRNAs according to *X* analyzed by FC or ***v***. **C** The receiver operating characteristic (ROC) curve of three analytic methods. The true positive rate is decided by essential genes and plotted against the false positive rate, decided by non-essential genes (left). The area under the curve analysis for essential (solid line) and non-essential (dotted line) genes (right). **D** The receiver operating characteristic (ROC) curve of CRISPR screening results of Brunello library using three analytic methods. **E** Calculated ROC-AUC values from **D**. **F** Recall percentage at 20% false discovery rate (FDR) calculated by three methods. **G** False discovery rate (FDR) recall at 95%, 97.5%, and 98% calculated by three methods

Simultaneous quantification of sgRNA frequency and activity can be used for correcting bias in screens with custom sgRNA library without the existing database of sgRNA activity or screening results across multiple cell lines (Fig. 1). As a proof of concept, we investigated whether our new analysis method can more accurately identify essential genes in a customized sgRNA library containing 6932 sgRNAs chosen from the Brunello library [15] targeting 2305 genes that are predicted to be druggable by small molecules [33] (Fig. 5A). NGS analysis revealed that most sgRNAs are well represented with 76% (5236/6932) and 98% (6793/6932) of sgRNA frequencies within library within twofold and fivefold difference from median frequency (0.12 reads per thousand), respectively (Additional file 1: Fig. S4A). Up to 80% of sgRNAs were correctly paired with their reporter sequences (Additional file 1: Fig. S4B). Variations between replicates were consistent regardless of sgRNA frequency in the library, indicating minimal genetic drift (Additional file 1: Fig. S4C, D). The fold change and indel frequency between biological replicates were well correlated (Pearson’s *r* = 0.81–0.83, *p* < 0.0001 for fold change and Pearson’s *r* = 0.88–0.90, *p* < 0.0001 for indel frequency; Additional file 1: Fig. S4E, F). The median indel frequency reached 77.9% 21 days after infection (Additional file 1: Fig. S4G), suggesting that a significant portion of the optimized sgRNAs did not edit 100% of their target genes. Low-specificity sgRNAs predicted by GuideScan [34] did not have lower fold change compared to high-specificity sgRNAs (Additional file: Fig. S4H). Remarkably, the*** v*** value obtained with the same method as Fig. 4 was much less dependent of indel frequency calculated as *X* (Fig. 5B). Similar to what was performed for tiling array screens, we first compared the effectiveness of conventional FC and*** v*** in identifying previously identified essential genes [35]. Similar to the results shown in Fig. 4H, the ROC-AUC value and dAUC were enhanced by 5.24% and 15.75%, respectively, with the*** v*** metric as the viability score (Fig. 5C). Our screening results analyzed with the*** v*** metric were more consistent with previous screening results in the DepMap project [36] compared to those with FC metric (Additional file 1: Fig. S5A). In contrast, JACKS analysis failed to increase ROC-AUC in our screens (Fig. 5C), possibly because of the difference in our library design containing reporter sequence.

Finally, we tested whether our analysis method and the indel frequencies analyzed in Fig. 4B, C can be applied to screening results generated by an independent group using the same library [32]. The*** v*** viability metric more efficiently identified essential genes with ROC-AUC reaching 0.9830, outperforming conventional FC analysis (0.9716) and JACKS analysis [25] (0.9771) (Fig. 5D, E, Additional file 1: Fig. S5B). Our analysis did not compromise accuracy (98.4%, 96.8%, and 96.8% at 20% false discovery rate with ***v***, FC, and JACKS, respectively) (Fig. 5F) while it was particularly effective in decreasing false negative data, with false discovery rate at 95% and 98% recall (true discovery) decreased by 2.0%p and 60%p compared to using FC values (Fig. 5G). This is expected as the essential genes represented in the library with low-activity sgRNAs, such as critical cell cycle regulator CDC25A [37, 38] in the present sgRNA library (median indel frequency of 37.7% 21 days after transduction), are classified as not essential (Additional file 1: Fig. S5C).

Our method also increased ROC-AUC in CRISPR screening results in 75% (12/16) of cell lines analyzed with an independent whitehead library [39, 40] (Additional file 1: Fig. S5D). The improvements were less dramatic compared to those in Fig. 5D–G because experimental conditions of our CRISPR screening would likely differ from those of previously published studies and therefore the sgRNA activity values are not accurate. Also, our analysis was limited to sgRNAs that are common in the libraries used in published results and those used in Fig. 5A (Additional file 1: Fig. S5D) [25, 39–41]. In fact, the whitehead library, which benefitted by using the*** v*** metric, had the largest number of common sgRNAs (1206 sgRNAs), whereas TKO (436 sgRNAs) and Yusa (1002 sgRNAs) libraries with fewer number of common sgRNAs failed to achieve a significant increase in ROC-AUC across cell lines. Our method showed comparable performance as JACKS across all libraries tested (Additional file 1: Fig. S5D).

### The new analysis method identified *NNMT*, *LDHB*, and *PTBP1* as vemurafenib resistance genes

**Fig. 6:**
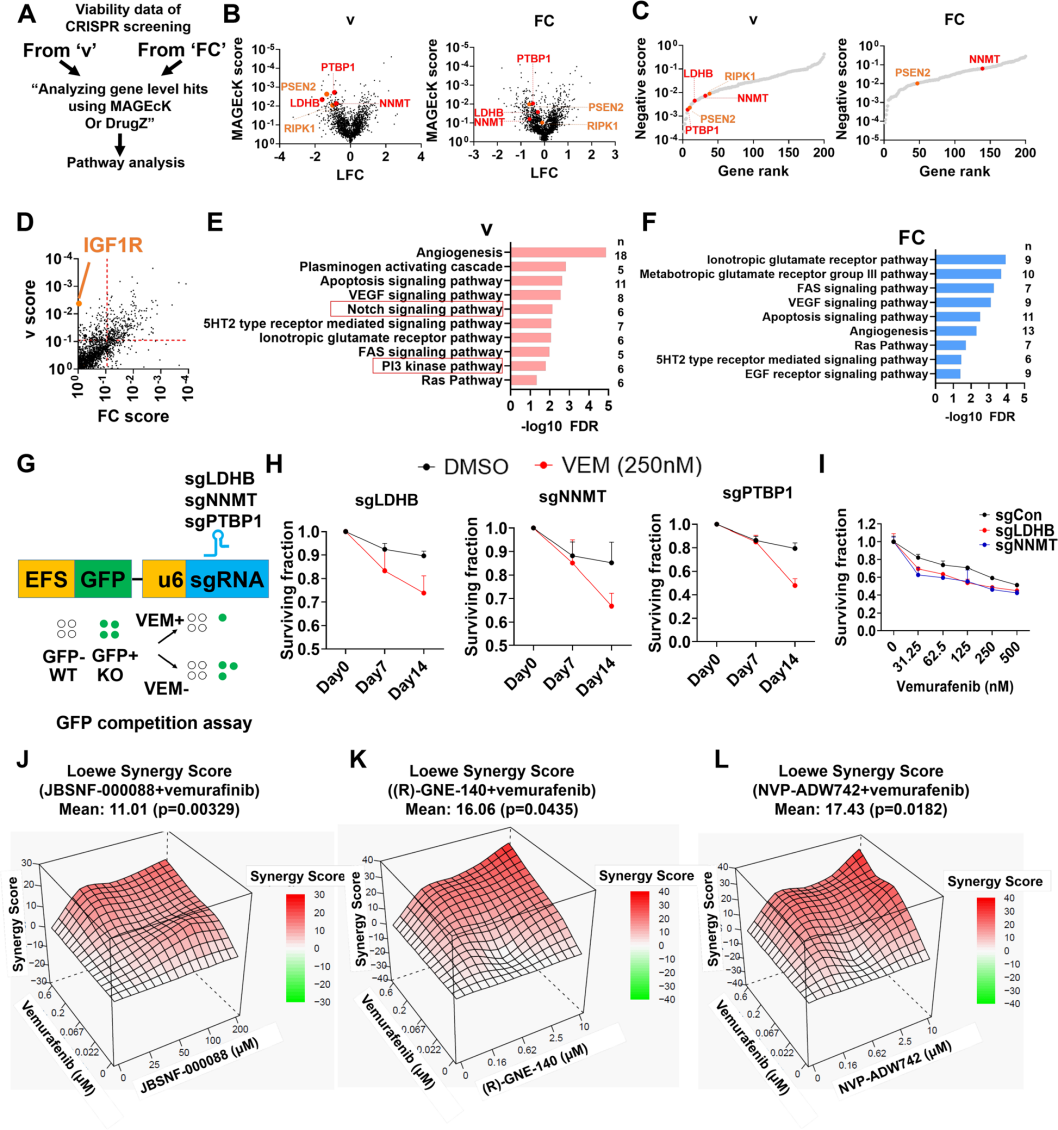
CRISPR-Cas9 screening using *v* metric prioritizes hits in A375 melanoma cells treated with vemurafenib. **A**–**C** MAGEcK results from the two analysis methods. **A** Schematic flow for analyzing hits from CRISPR-Cas9 screening using two methods. **B** Volcano plots of MAGEcK results using ***v*** (left) and FC metrics (right). **C** Top rank genes were selected through the MAGEcK score using ***v*** (left) and FC metrics (right). **D** Comparison of the MAGEcK score of hits between ***v*** and FC. **B**–**D** Orange color genes were previously reported as synergistic genes with vemurafenib while red color genes were newly identified in this study. **E**, **F** Pathway analysis results using Panther pathway overrepresentation test (score < 0.09 from **E**) using **E v** and **F** FC. **G** Schematic figure of GFP competition assay. **H** GFP competition assay with GFP-labeled A375 cells ablated of indicated genes in the presence of vemurafenib. **I** Proliferation assay of A375 cells ablated of indicated genes. All data are presented as mean ± s.d. *n* = 3 biological replicates. **J**–**L** Drug synergy score calculated by SynergyFinder in A375 cells treated with indicated drugs in combination with vemurafenib

sgRNA activity-corrected viability profile could be used for more efficient identification of genes with the desired phenotype. Hence, we analyzed CRISPR screens in Fig. 5A using conventional FC or*** v*** as metrics of viability in the presence and absence of the BRAF inhibitor, vemurafenib [42], to identify genes whose ablation could sensitize melanoma cells to vemurafenib. Both FC and*** v*** values were used for MAGeCK analysis [43] to calculate the gene-level significance of essentiality in the presence and absence of the drug (Fig. 6A–F). The PANTHER overrepresentation test [44, 45] revealed that the Notch signaling and PI3 kinase pathways (Fig. 6E, F), which are known to be involved in BRAF or MEK inhibitor resistance in melanoma [46–51], were exclusively identified in the list of hits obtained by analyzing ***v***. In addition, the MaGECK [43] analysis using*** v*** identified *NNMT*, *LDHB*, *PTBP1*, receptor-interacting serine/threonine-protein kinase 1 (*RIPK1*), insulin-like growth factor 1 receptor (*IGF1R*), and presenilin-2 (*PSEN2*) as among the top hits that were not identified by conventional analysis with FC (Fig. 6A–C). *IGF1R* [52], *RIPK1* [53], and *PSEN2* [54] are already known to contribute to vemurafenib resistance, suggesting that our new analysis method using*** v*** reliably identified hits whose ablation can sensitize melanoma to vemurafenib. We similarly used FC and*** v*** values as input for the DrugZ analysis [55] (Fig. 6A). *LDHB*, *RIPK1*, and *NNMT* were similarly more effectively identified as hits using ***v*** values for the DrugZ analysis. TANK-binding kinase 1 (*TBK1*) [56, 57] and fibroblast growth factor 2 (*FGF2*) [58–60], which were previously reported to be involved in vemurafenib resistance, were also identified as top hits by using DrugZ with*** v*** input (Additional file 1: Fig. S6).

*NNMT* [61–63], *LDHB* [64, 65], and *PTBP1* [66–68] have been studied for promoting cancer development, but their synergistic effects with vemurafenib are not clear. To validate the effect of *NNMT*, *LDHB*, and *PTBP1* in A375 cells with vemurafenib treatment, we performed a GFP competition assay, where the sgRNA-expressing cells labeled with GFP were cocultured with unlabeled, wild-type cells, and the relative viability of sgRNA-expressing cells was analyzed by changes in the frequency of GFP-positive cells (Fig. 6G, H). When each of the three genes was knocked out by CRISPR-Cas9, the GFP-guide RNA-expressing cells selectively lost their fitness in the presence of vemurafenib (Fig. 6I). We further confirmed that the *NNMT* or *LDHB* knockout (Additional file 1: Fig. S7) reduced the survival rate when compared to wild-type cells under vemurafenib treatment (Fig. 6H). Finally, JBSFN000088, (R)-GNE-140, and NVP-ADW742 which are inhibitors of NNMT, LDHB, and IGF1R respectively, showed a synergistic effect with vemurafenib compared to the DMSO-treated control group (Fig. 6J–L, Additional file 1: Fig. S8). In addition, these inhibitors overcame resistance in vemurafenib-resistant A375-VR cells and Hs294T cells, which are intrinsic BRAF inhibitor-resistant cell lines [69] (Additional file 1: Fig. S9, 10). Collectively, the new analysis using*** v*** effectively identified potential therapeutic target genes whose inhibition can overcome resistance to targeted therapy.

## Discussion

Pooled CRISPR screens enable a massive parallel inquiry of phenotypic changes upon ablation of thousands of genes in one experiment. Although this method is robust and economical, inherent noise and bias is stemming from the fact that the behaviors of individual cells ablated with each gene cannot be observed. Our work provides a widely applicable method to perform genetic screening and identify hits with a substantially reduced risk of bias due to variations in sgRNA activity. Especially, we successfully implemented our method to previous CRISPR screening results for better identification of essential genes. This highlights the generalizability of our approach.

Our method is unique as we used actual, measured sgRNA efficiency estimate to correct the screening results whereas other approaches were intended to use sgRNA efficiency estimates inferred from screening results generated in previous studies. Therefore, our method is best suited for screens using custom sgRNA libraries with little to no available published screening results to infer sgRNA activities. Our method is also easily applicable to screens using other Cas nucleases such as Cas12a and *Campylobacter jejuni* Cas9 (CjCas9) and *Streptococcus canis* Cas9 (ScCas9) [20, 70], with much less options in sgRNA activity optimizations. The increasing number of sgRNAs per gene in the library can improve the quality of the CRISPR screens by minimizing false negative results. However, especially for genomewide screens, the scales of the sgRNA library and subsequent screens grow too large with this approach. Our method can be used to downsize the sgRNA library by adjusting the viability phenotype of low-activity sgRNAs and minimizing false negatives with less number of sgRNAs. This could particularly be useful for CRISPR screens performed with complex models such as primary cultures with limited scalability.

Intriguingly, the presence of a mutation in the reporter sequence was tightly coupled to that of the endogenous sgRNA target gene at the single-cell level. This is likely a consequence of the variation in the expression of the lentivirally delivered Cas9 gene and sgRNAs. Cells with edited reporter sequences are likely to have a higher expression of Cas9 and sgRNAs than those with intact reporter sequences. Thus, cells with edited reporter sequences are also more likely to have an endogenous target gene edited.

Finally, our new analysis method identified NNMT and LDHB, which would otherwise be missed in conventional analysis, as novel target genes whose ablation sensitizes melanoma cells to vemurafenib. One of the mechanisms of BRAF inhibition is the induction of apoptosis [71]. NNMT1 reinforces chemoresistance by stabilizing the SIRT1 protein [63], which plays a crucial role in cancer drug resistance, including apoptosis inactivation [72, 73]. In addition, inhibiting LDHB increases apoptosis in cancer cells [64], in combination with chemotherapy [74]. Further studies are needed to identify the detailed molecular mechanisms underlying the role of the novel target genes in promoting resistance to therapy.

There are several sources of inaccuracies in measuring sgRNA activity with reporter sequence edit. The editing efficiency by sgRNA does not only depend on the sequence of sgRNA but also by the chromatin landscape of the target sequence [75]. Our reporter sequence as part of a lentiviral transgene integrated at random sites within genome cannot have the same chromatin landscape as the endogenous target gene. In fact, while the indel frequencies of the reporter sequence and the endogenous target gene are correlated, the values were not identical (Fig. S1E). Also, our reporter sequence cannot detect moderate to large size (> 10nt towards the 5′ end of the sgRNA sequence) deletion. This was inevitable as the distance between the sgRNA and the reporter sequence should be minimal for maximal efficiency in PCR amplification of the sgRNA-reporter cassette for NGS, and to prevent decoupling of the sgRNA and the reporter sequence. However, recent studies showed that the size of the indel mutation is non-random and can vary with sgRNA [76]. Therefore, varying fraction of indel mutations in the reporter sequence for each sgRNA may have been lost in the analysis, contributing to bias in the analysis. Finally, the sgRNA and reporter sequence can be coupled at a higher rate than achieved in our screens (Figs. S1A, S4A) by minimizing the physical distance between sgRNA and the reporter sequence, reducing PCR cycles and extending elongation time during library construction and NGS amplicon generation, and decreasing transfection rates [27]. Further modifications for maximizing the accuracy of reporter sequence as a proxy for target gene edit in our analysis may significantly improve the CRISPR screening results.

Although our analysis revealed that the differences in sgRNA activity are the major determinant of variations in viability in CRISPR screens, this does not exclude the possibility that the other attributes of sgRNAs contribute to the bias in screens. It is likely that the effects of other attributes are masked by the sgRNA activity effect. For example, the off-target effect of sgRNA can still be a contributing factor to the noise in the screens. The use of high-fidelity variant Cas9 [21, 23, 77] and specificity-optimizing tools such as GuideScan [34] can be used in combination with our library design for optimal performance. Also, the functional importance of the sgRNA target site within a gene can influence the viability phenotype. Xu and colleagues reported that in frame mutations, which are generally considered neutral, targeting essential protein domain can abolish protein function thereby forming CRISPR knockout hypersensitive regions [78]. As previous studies showed that a significant fraction of indel mutations are in frame [76], designing sgRNAs to maximize the essentiality of the sgRNA targeting site within the gene will significantly improve the robustness of the phenotype.

Other sources of noise can originate from population drift or off-target effects. Our method for determining*** v*** relies on identifying the relative FC values within the window of FC values expected when*** v*** is equal to 0 and 1. However, the expected values of FC_***v***=1_ and FC_***v***=0_ converge with low *X* values, so a small noise in FC can be overrepresented as a large fluctuation in *v*. Therefore, we believe that our analysis will be particularly useful in combination with on-target activity-optimized sgRNA libraries with high *X* values.

## Conclusions

We develop and validate the method to adjust the phenotype scores with measured sgRNA activity in CRISPR viability screens. We expect our method can be used for broad applications where options for sgRNA optimization are limited.

## Methods

### Cell culture

HEK-293 T cells were maintained in Dulbecco’s modified Eagle’s medium (DMEM) supplemented with 10% fetal bovine serum (Welgene) and antibiotics. A375, Hs294T cells were maintained in RPMI1640 supplemented with FBS and antibiotics. All cell lines were obtained from Korean Cell Line Bank (https://cellbank.snu.ac.kr).

### Library construction

Oligonucleotide pools synthesized by Twist Biosciences were cloned into the hU6 destination vector with Gibson assembly. The number of PCR cycles was minimized (~ 10 cycles) and extension time lengthened (5 min per kilobase) to maximize the sgRNA-reporter coupling rate [79]. The resulting hU6-sgRNA cassette was subcloned to FUW-EFS-BlastR lentiviral plasmid. The tiling-array library contained 1126 gRNAs for seven genes (RPL8, RPL15, WWTR1, FNTA, GSK3B, CCR5, and CD4) and the druggable gene library contained 6935 gRNAs targeting 2305 genes. All library vectors included the reporter target sequences that could be cleaved by the gRNA expressed in the same vector. All library constructions were done at a fold coverage of at least 500 to preserve the diversity of the sgRNAs within the library.

### Viral vector transfection and virus production

The lentiviral sgRNA library plasmid was co-transfected with packaging vectors psPAX2 and pVSV-G (kind gifts from the lab of Timothy Lu) into HEK-293 T cells using PEI Max (Polyscience, Inc.). The lentiviral supernatant was collected 48 h after transfection, cleared of contaminating HEK-293 T cells, and stored at − 80 °C. To obtain appropriate lentiviral titer, A375-Cas9 cells were infected with twofold serial dilutions of lentivirus, and cells were grown in 96-well plates in the absence and presence of blasticidin. The fraction of infected cells were calculated by the relative viability of cells treated with blasticidin compared to those not treated.

### Viral transduction into A375 cells

The lentiviral sgRNA library was delivered into A375-Cas9 cells stably expressing Cas9 at a multiplicity of infection (MOI) of 0.3–0.5, using 8 µg/mL polybrene. Two days later, the A375 cells were treated with blasticidin for 3 weeks until A375 cells were harvested. For the druggable gene library screens, A375 cells were treated with vemurafenib (MedChemExpress) or DMSO. Cells were passaged with fold coverage of at least 500 every 2 or 3 days.

### Genomic DNA extraction and next-generation sequencing (NGS)

Genomic DNA was extracted using Accuprep genomic DNA extraction kit (Bioneer), according to the manufacturer’s instructions. The PCR amplicon spanning the sgRNA and reporter sequence was generated using primers.

F: 5′-CAAGCAGAAGACGGCATACGAGATNNNNNNGGACTATCATATGCTTACCGTAACTTG-3′ R: 5′-AATGATACGGCGACCACCGAGATCTACAC AAGCAGCGTATCCACATAGC-3′. Deep sequencing was performed using HiSeq2500 at a 100 nucleotide read length paired end. The primers used for sequencing were as follows (5′-3′): Read1 (reading the reporter sequence): CGTCAGGAATTATCCGGTGCCTAGAGAAGGTCC, Read2 (reading the sgRNA): CCGTAACTTGAAAGTATTTCGATTTCTTGGCTTTATATATCTTGTGGAAAGGACGAAACACCG, and Index: CGTCCTTTCCACAAGATATATAAAGCCAAGAAATCGAAATACTTTCAAGTTACGGTAAGCATATGATAGTCC.

### 6-TG treatment in HPRT1 knockout cells

Four guide RNAs targeting HPRT1 were cloned into lentiviral vectors and used to construct the lentiviruses. Each constructed vector contained HPRT1 reporter sequences targeted by each HPRT1 guide RNA to check the indel frequency rates of the reporter sequences. Each virus was transduced into A375 cells and 10 µg/mL blasticidin (Invivogen) for 1 week for selection. Then, 6-TG or DMSO was added for 2 weeks until the cells were harvested for genomic DNA extraction. The genomic locus flanking the sgRNA target sequence and the reporter sequence were PCR amplified with Q5 High-Fidelity DNA polymerase (New England Biolabs). The adapters for deep sequencing were appended to the PCR amplicons using xGen DNA library prep kits (Integrated DNA Technologies) for NGS analysis. The presence of indel mutations in the HPRT1 locus and the reporter sequence were quantified using CRISPRESSO2 [80].

### Calculating actual indel frequency *X* and new phenotype score ***v***

The frequencies of guide RNAs and their respective indel frequencies were counted using custom code available in github (https://github.com/tackhoonkim). The resulting read counts were subject to analysis as described below with Microsoft Excel.

As noted in Fig. 4F, the non-targeting control sgRNA frequency changed with its target cleavage efficiency. Therefore, the normalized fold change of each sgRNA frequency, FC_norm_, was calculated as:   

$$FC_{norm}=FC_{raw}\div FC_0$$

where FC_0_ is the fold change of a theoretical control sgRNA of zero target DNA cleavage activity. FC_0_ was obtained by linear regression of fold change and indel frequency of control sgRNAs (Fig. 4F). 

 Subsequent calculation of adjusted indel frequency *X* and viability metric*** v*** was done as described in the “Results” section. Briefly, the adjusted, “actual” indel frequency *X* was calculated as:

$$X=1-{\mathrm{FC}}_{\mathrm{norm}}+{\mathrm{FC}}_{\mathrm{norm}}\times \mathrm{indel}$$

where indel is the “observed” indel frequency of obtained from the NGS analysis.

Next, adjusted, actual viability*** v*** of cells with sgRNA target gene disruption was calculated as:

$${\varvec{v}}=\frac{{\mathrm{FC}}_{\mathrm{norm}}-{\mathrm{FC}}_{{\varvec{v}}=0}}{{\mathrm{FC}}_{{\varvec{v}}=1}-{\mathrm{FC}}_{{\varvec{v}}=0}}=\frac{{\mathrm{FC}}_{\mathrm{norm}}-\left(1-X\right)}{{\mathrm{FC}}_{{\varvec{v}}=1}-\left(1-X\right)}$$

where FC_***v***=*0*_ is a theoretical FC value given zero viability upon gene disruption and indel frequency *X* and is equal to 1 − *X*. FC_***v***=1_ is a theoretical FC value given no change in viability (***v*** = 1) upon gene disruption and was obtained as an equation for linear regression of FC_norm_ and *X* for non-targeting control sgRNAs (Fig. 4F).

MAGEcK analysis was done as previously described [43]. The sgRNA count data for the day 21 sample for input data were generated by multiplying either FC or*** v*** to the initial sgRNA count in the plasmid. Subsequent procedures were followed by the user instruction (https://sourceforge.net/p/mageck/wiki/Home/). DrugZ [81] was performed following user instruction (https://github.com/hart-lab/drugz).

To apply our approach to previous works, CRISPR screening results as raw sgRNA counts were obtained from previous studies by Doench and colleagues [15] and Parts and colleagues [25], for identical analysis to obtain *X* and ***v***. The sgRNA activities obtained in Fig. 5A are directly applied to the data. The fold changes of replicates were averaged. JACKS analysis was performed as instructed in https://github.com/felicityallen/JACKS.

### GFP competition assay

The gRNAs for each hit gene were cloned into GFP-expressing lentiviral vectors FUW-EFS-GFP. Five days after virus transduction into A375 cells stably expressing Cas9, the fraction of GFP-positive cells was measured using BD Accuri C6Plus as the initial fraction of knockout cells. The same quantification of GFP-positive cell fraction was done 7 and 14 days after the initial flow cytometry experiment. The relative viability of GFP-positive knockout cells at “day x” relative to GFP-negative wild-type cells was calculated as below:

$$\mathrm{Relative\;viability}=\frac{{}^{{\mathrm{GFP}}_{\mathrm{day }x}^{+}}\!\left/ \!{}_{{\mathrm{GFP}}_{\mathrm{day }x}^{-}}\right.}{{}^{{\mathrm{GFP}}_{\mathrm{initial}}^{+}}\!\left/ \!{}_{{\mathrm{GFP}}_{\mathrm{initial}}^{-}}\right.}$$

### Pathway enrichment analysis

After discovering hits using MAGEcK, we performed pathway analysis using the PANTHER overrepresentation test with selected genes (genes from each method, score < 0.09).

### Cell viability assay with chemical inhibitors

A375 and A375-VR cells (1000 cells/well) and Hs294T cells (2000 cells/well) were seeded in 96-well plates. The next day, inhibitors and vemurafenib were treated at indicated concentrations. The WST assay was performed using EZ-cytox reagent (DogenBio) diluted in RPMI1640. The absorbance at the 450-nm wavelength was measured using Wallac EnVision (Perkin Elmer) 3 days after drug treatment. Drug synergies were evaluated by performing SynergyFinder (https://synergyfinder.org) [82].

### Statistical analysis

Data are presented as mean ± s.d. Correlations were evaluated using Pearson’s correlation coefficient *r* analyzed by GraphPad Prism. One-sample Wilcoxon test and two-tailed test were used to calculate the *p* value.

## Supplementary Information

**Additional file 1:** **Figure S1.** Data quality control of Tiling array library screens. **Figure S2.** Indel frequency adds significant bias to log fold change (LFC) of gRNA frequency. **Figure S3.** Correlation of indel frequency and conventional FC reveals bias in phenotype score. **Figure S4.** Quality control of druggable gene CRISPR screen data in Figures 5 and 6. **Figure S5.** Comparison of our method to previous approaches. **Figure S6.** Effective synergistic targets with vemurafenib treatment using DrugZ with *v *as input. **Figure S7.** Confirmation of ablation of target genes by T7 endonuclease assay for Figure 6H. **Figure S8.** Dose response matrix data for Figures 6I-K. **Figure S9.** Drug synergy data for A375 VR cells. **Figure S10.** Drug synergy data for Hs294T cells.

## Data Availability

All data generated or analyzed during this study are included in this published article, its supplementary information files, and publicly available repositories. The NGS data for CRISPR screens in Figs. 2 and 5 are available in NCBI SRA (PRJNA856494). The numerical data for all the plots in the main and supplementary figures are in FigShare (https://doi.org/10.6084/m9.figshare.21971246). The codes used for the NGS or sgRNA count analysis are provided in the links below: sgRNA counter and indel detector (custom code): https://github.com/tackhoonkim (Zenodo repository DOI: https://doi.org/10.5281/zenodo.7575490). MAGeCK: https://sourceforge.net/p/mageck/wiki/Home/ JACKS: https://github.com/felicityallen/JACKS DrugZ: https://github.com/hart-lab/drugz Synergy Finder: https://synergyfinder.org

## Abbreviations

NNMT: Nicotinamide N-methyltransferase
LDHB: Lactate dehydrogenase B
UMI: Unique molecular identifier
6-TG: 6-Thioguanine
PTBP1: Polypyrimidine tract-binding protein 1
RIPK1: Receptor-interacting serine/threonine-protein kinase 1
IGF1R: Insulin-like growth factor 1 receptor
PSEN2: Presenilin-2
TBK1: TANK-binding kinase 1
FGF2: Fibroblast growth factor 2
CjCas9: *Campylobacter jejuni* Cas9
ScCas9: *Streptococcus canis* Cas9
NCBI SRA: National Center for Biotechnology Information Sequence Read Archive
