# Supplementary material for: Efficient prioritization of CRISPR screen hits by accounting for targeting efficiency of guide RNA
Source: BMC Biol. 2023 Feb 24;21:45. doi: 10.1186/s12915-023-01536-y (PMC9960226; doi:10.1186/s12915-023-01536-y)
Supplement: Supplementary file 1 — Additional file 1: Figure S1. Data quality control of Tiling array library screens. Figure S2. Indel frequency adds significant bias to log fold change (LFC) of gRNA frequency. Figure S3. Correlation of indel frequency and conventional FC reveals bias in phenotype score. Figure S4. Quality control of druggable gene CRISPR screen data in Figures 5 and 6. Figure S5. Comparison of our method to previous approaches. Figure S6. Effective synergistic targets with vemurafenib treatment using DrugZ with v as input. Figure S7. Confirmation of ablation of target genes by T7 endonuclease assay for Figure 6H. Figure S8. Dose response matrix data for Figures 6I-K. Figure S9. Drug synergy data for A375 VR cells. Figure S10. Drug synergy data for Hs294T cells. [file 12915_2023_1536_MOESM1_ESM.docx]

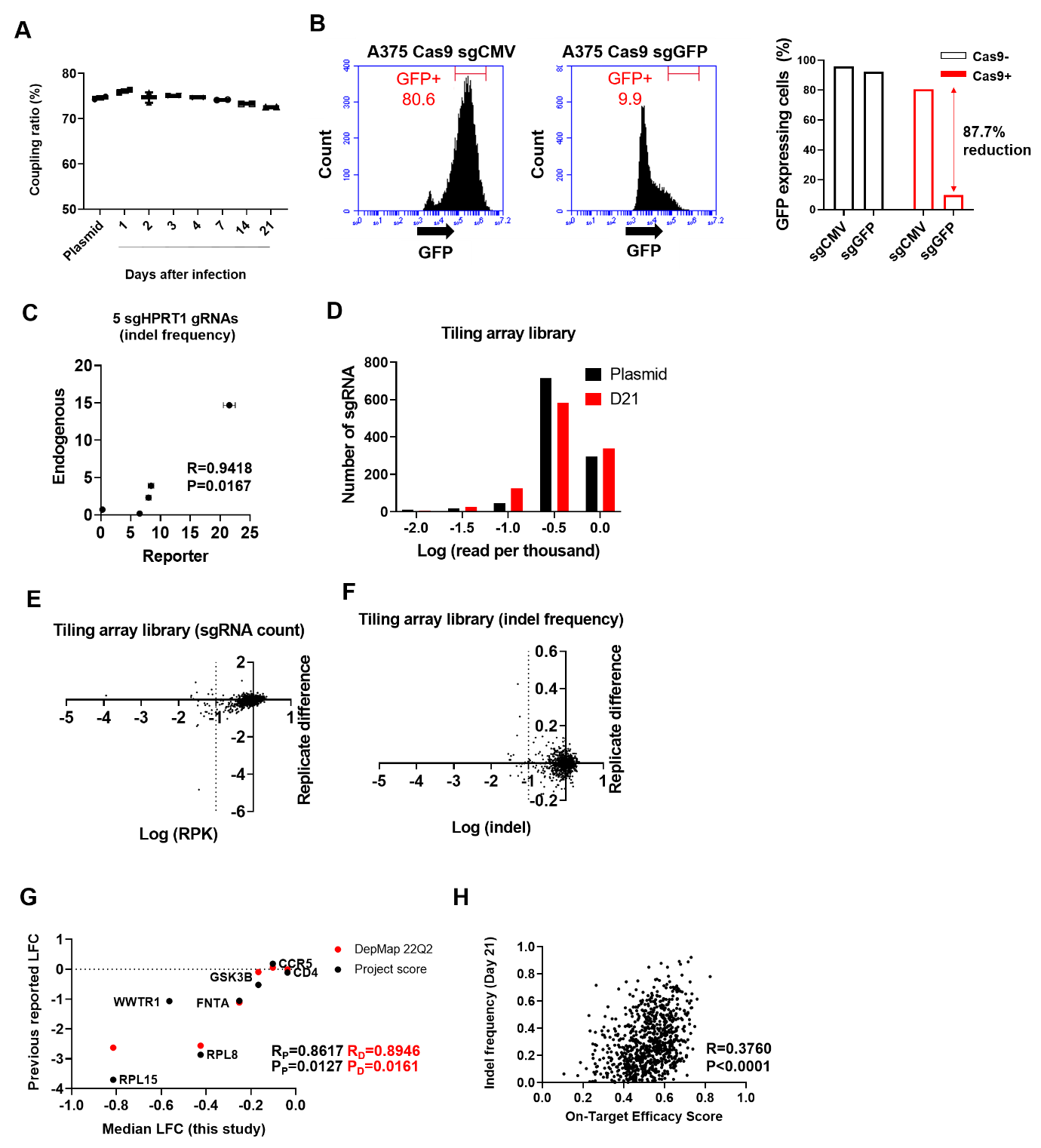


**Fig. S1** **Data quality control of Tiling array library screens. A** Coupling ratio between the gRNA and reporter sequence targeted by the same gRNA from each group. Data are presented as mean ± s.d. **B** Validation of Cas9 activity of the A375-Cas9 cell used in this study. GFP was expressed with sgGFP or negative control sgCMV. Successful indel mutation of GFP results in decrease in GFP+ populations. **C** Correlation between indel frequency of reporter sequence and those of endogenous target sequence for five sgRNAs targeting HPRT1 gene. **D** Histogram of the frequency of sgRNA used in Tiling array library. **E-F** sgRNA frequency in library plasmid was compared to the **E** Differences in log fold changes between biological replicates in tiling array library screen, and **F** Differences in indel frequencies between biological replicates in tiling array library screen. **G** Comparison of log-fold changes between median LFC from this study and Project Score from Sanger Institute or DepMap score from Broad institute. **H** Correlation of on-target efficacy score calculated by CRISPick software of Broad Institute and indel frequency (day 21) from this study. For all figures, R indicates Pearson correlation coefficient r and p value is calculated by two-tailed test.


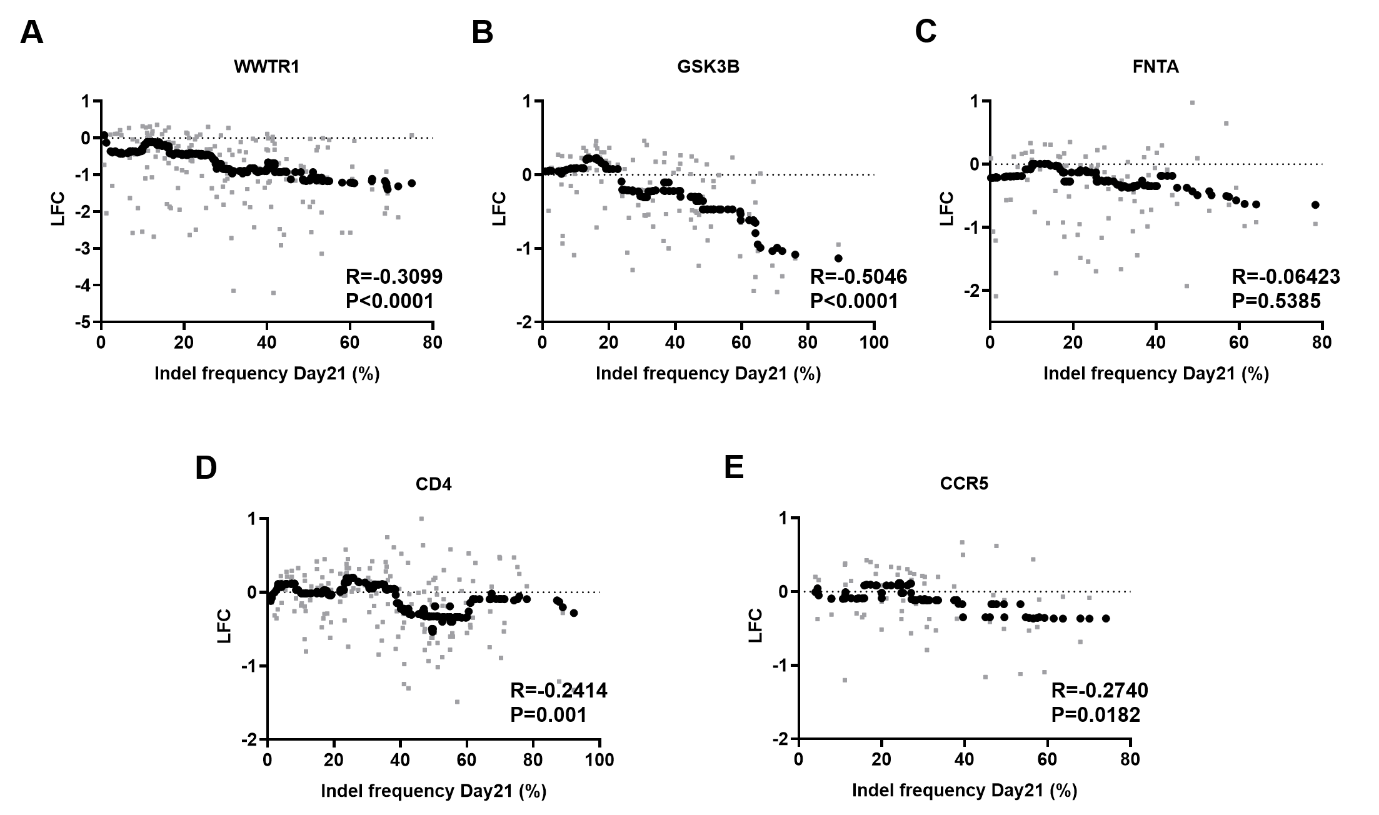


**Fig. S2 Indel frequency adds significant bias to log fold change (LFC) of gRNA frequency.** **A-E** Correlation between indel frequency (day 21) and moving median of LFC of each gene analyzed in Tiling array library screen. **A** WWTR1. **B** GSK3B. **C** FNTA. **D** CD4. **E** CCR5. Gray dots indicate values for each individual sgRNA while black bold dots indicate the moving median of 20 nearest neighbors. R indicates Pearson correlation coefficient r and p value is calculated by two-tailed test..


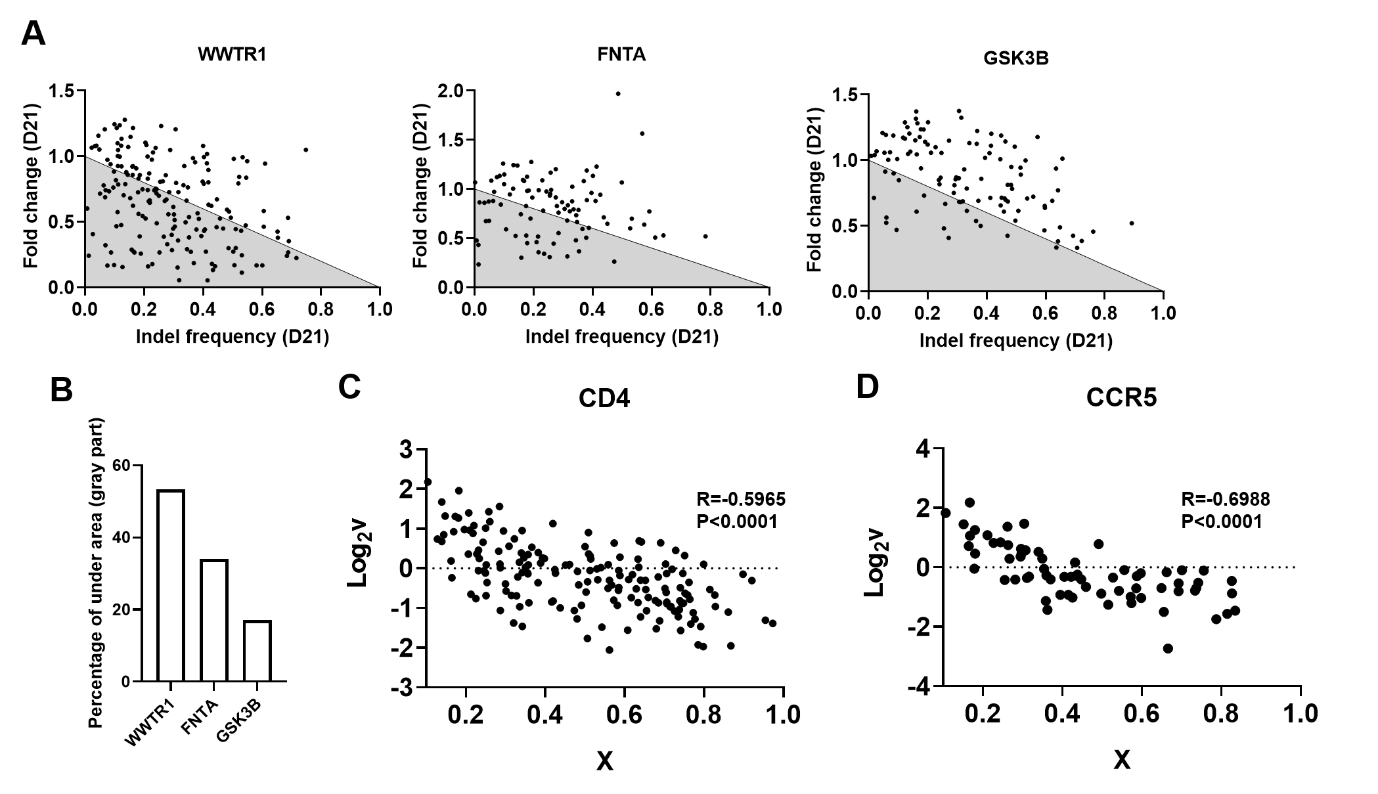


**Fig. S3** **Correlation of indel frequency and conventional FC reveals bias in phenotype score.** **A** Scatter plots for indel frequency and fold change at day 21 for sgRNAs targeting indicated genes. The black line indicates the theoretical minimum FC at viability = 0. Gray shade indicates an area below the theoretical minimum FC. The black line indicates the expected line at viability = 0. **B** Quantification of cells at expected viability below 0 from (**A**) (gray). **C-D** Scatter plots of X and log_2_***v*** for indicated genes. R means Pearson correlation coefficient r and p value is calculated by two-tailed test.


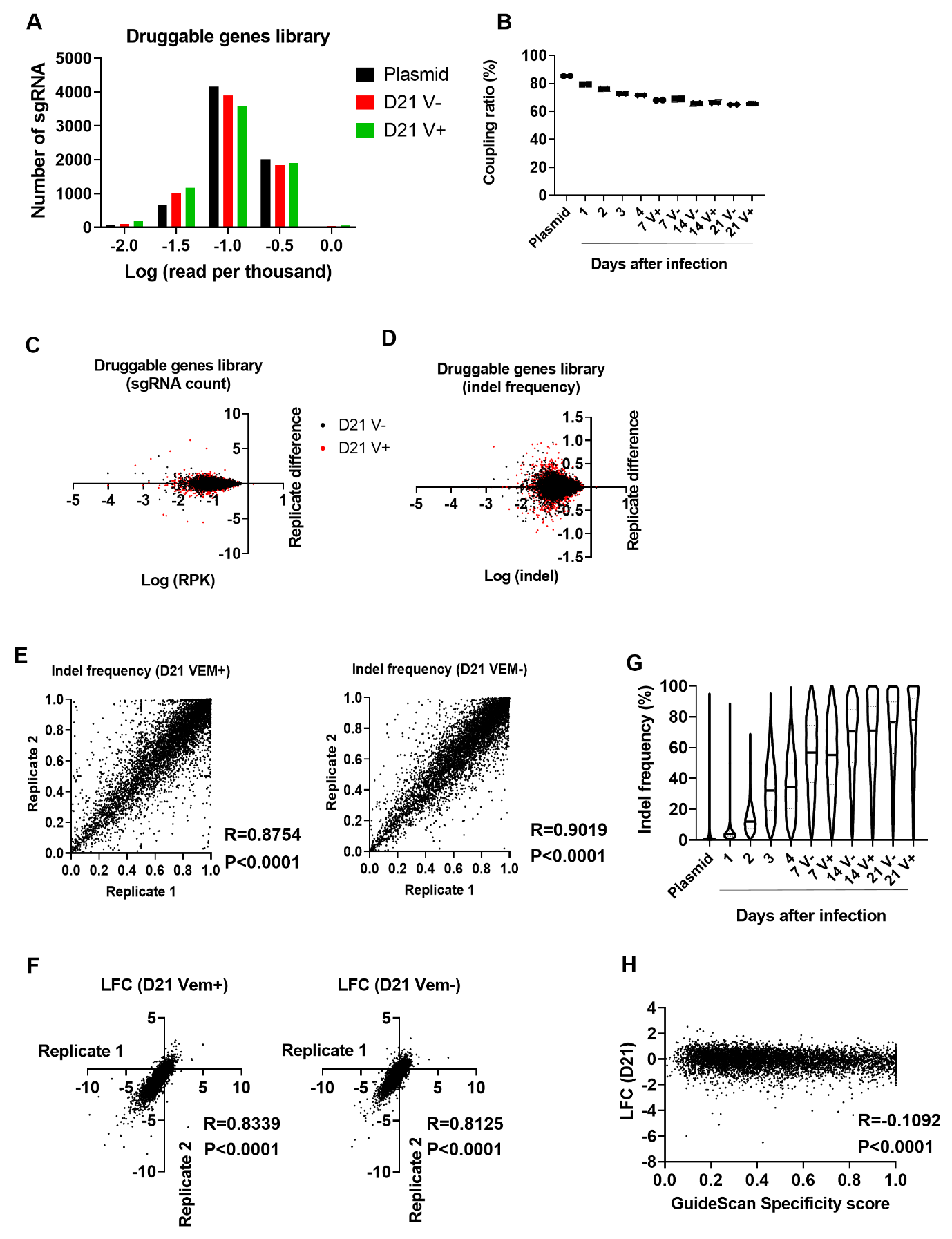


**Fig. S4** **Quality control of druggable gene CRISPR screen data in Figures 5,6.** **A** Histogram of frequencies of sgRNAs in the druggable gene sgRNA library used in Figures 5, 6. **B** Coupling ratio between the gRNA and reporter sequence targeted by the same gRNA from each group. Data are presented as mean ± s.d. **C-D** sgRNA frequency in library plasmid was compared to the **C** Differences in log fold changes between biological replicates and **D** Differences in indel frequencies between biological replicates. **E-F** Scatter plots showing the correlation between **E** indel frequencies and **F** log-fold change of two replicates (VEM+: vemurafenib treated replicates; VEM-: DMSO treated replicates). R indicates Pearson correlation coefficient r and p values are calculated by two-tailed test. **G** Indel frequencies of Druggable genes library from the indicated groups. Bold line indicates the median indel frequency **H** Scatter plots for GuideScan specificity score and fold change at day 21. R indicates Pearson correlation coefficient r and p value is calculated by two-tailed test.


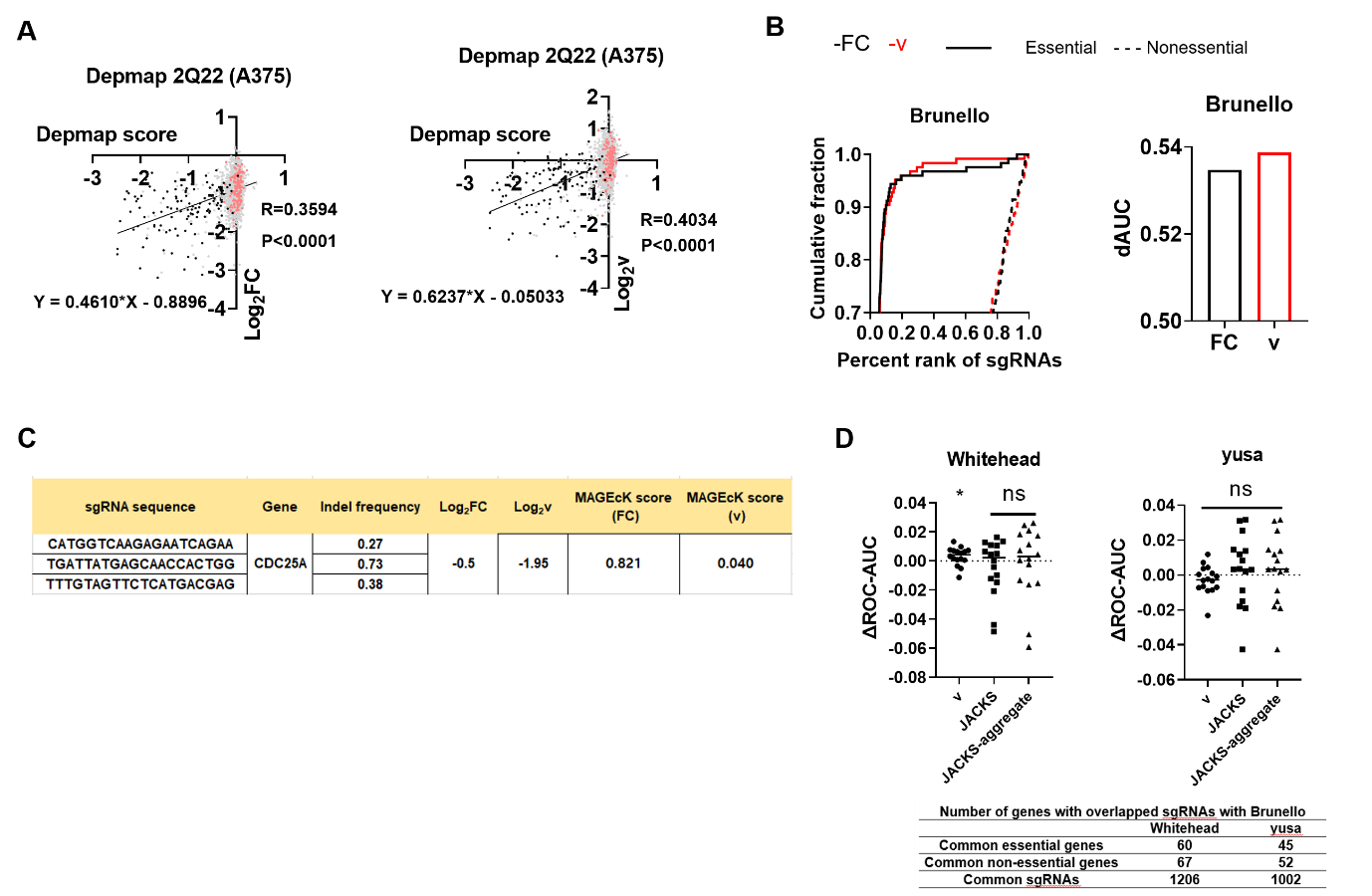


**Fig. S5 Comparison of our method to previous approaches.** **A** Comparison between log fold changes and DepMap score for screens done in figure 5. R indicates Pearson correlation coefficient r and p values are calculated by two-tailed test. Pink dots indicate non-essential genes while black dots indicate essential genes. **B** (left) AUC analysis of CRISPR screening results used in (fig 5D) for essential (solid line) and non-essential (dotted line) genes. (right) Calculated dAUC values. **C** raw data of FC and ***v*** values for CDC25A.**D** Change in ROC-AUC values compared to FC analysis with ***v*** analysis and JACKS for two independent libraries. “JACKS” analysis applied data from each cell line separately while “JACKS-aggregate” applied data from all cell lines as one set. Asterisk indicates P < 0.05, and p values were calculated using one-sample Wilcoxon tests.

**
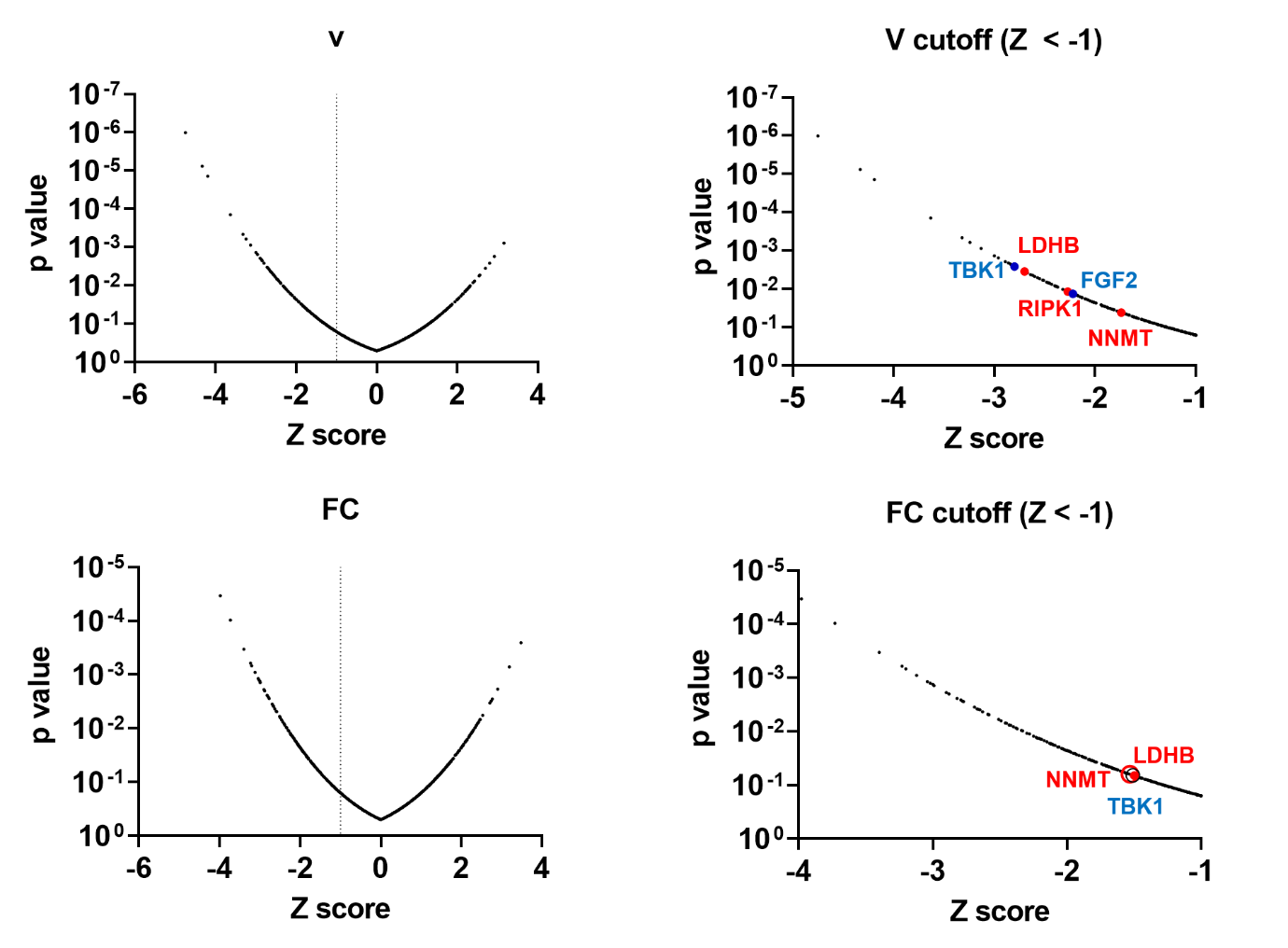
 Fig. S6** **Effective synergistic targets with vemurafenib treatment using DrugZ with *v* as input.** In ***v*** method, effective targets with vemurafenib treatment were revealed as high-rank hits.

**
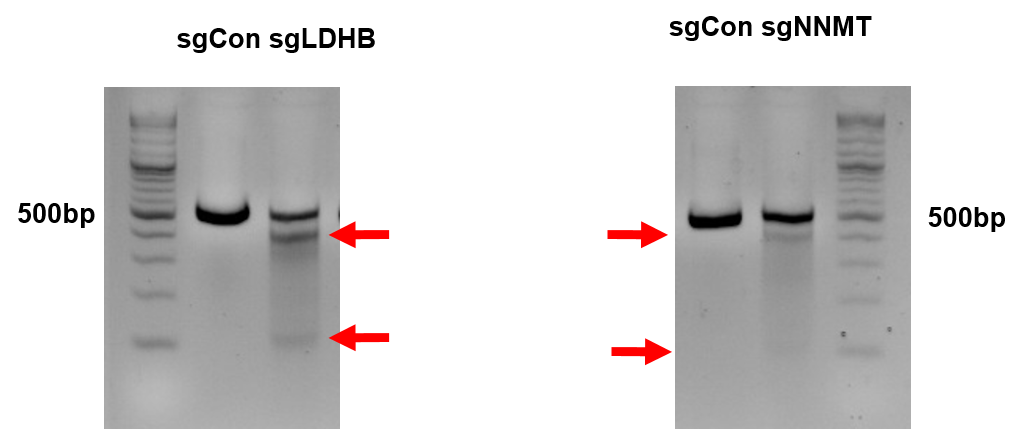
**

**Fig. S7.** **Confirmation of ablation of target genes by T7 endonuclease assay for Figure 6H.** Red arrows indicate DNA fragments generated by T7 endonuclease in the presence of indel mutation.


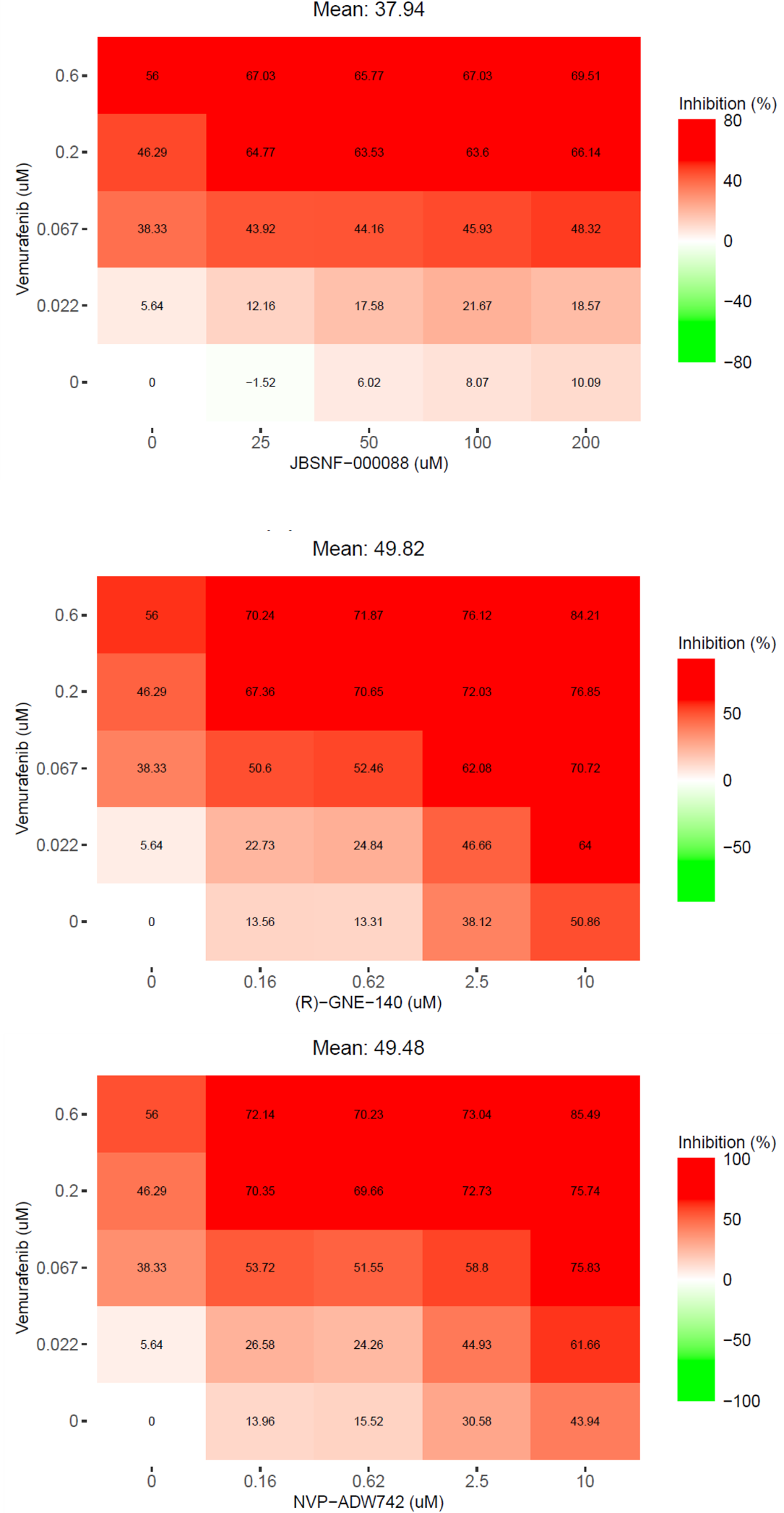


**Fig. S8 Dose response matrix data for Figures 6I-K**

**
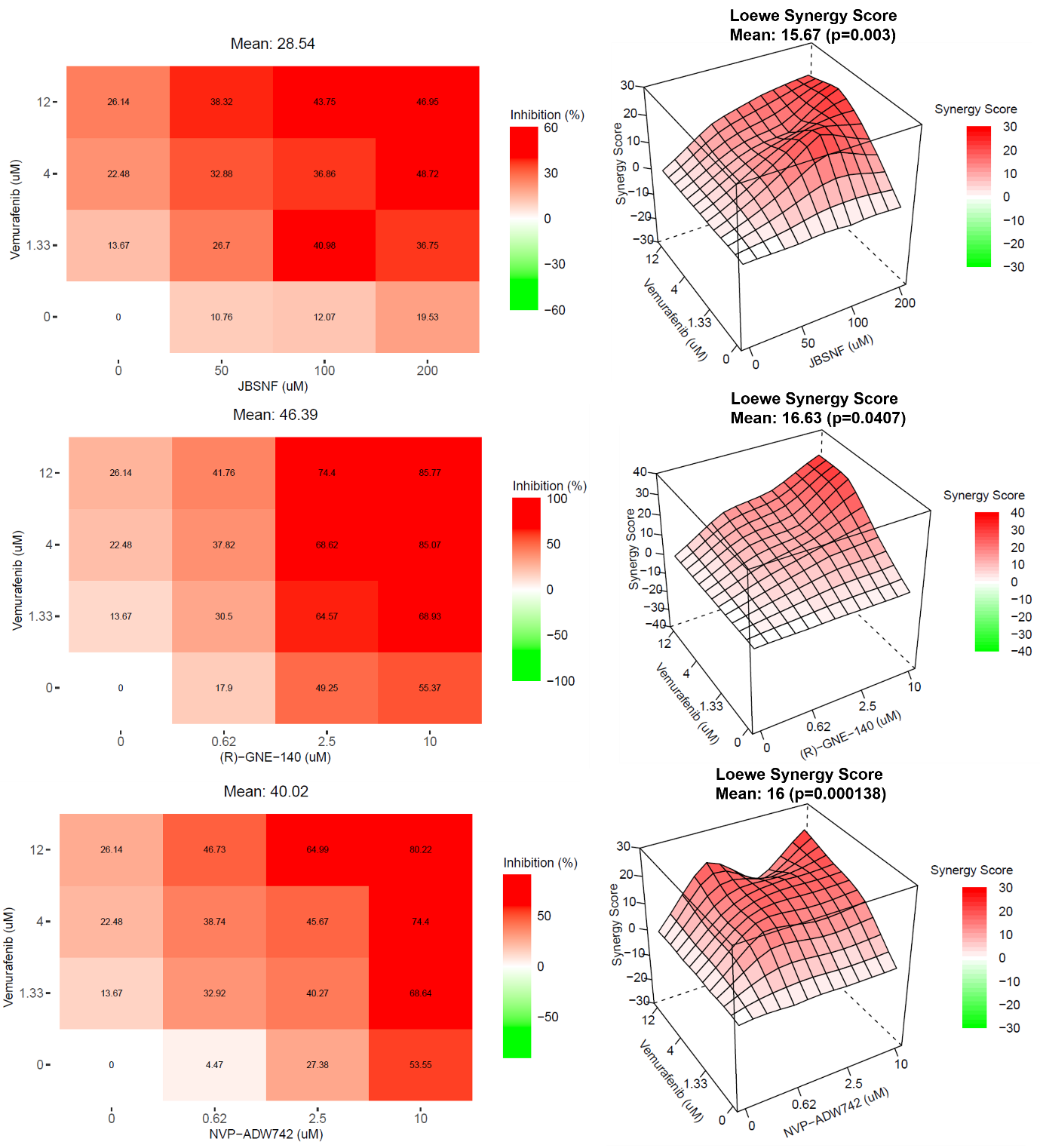
**

**Fig. S9 Drug synergy data for A375 VR cells**

**
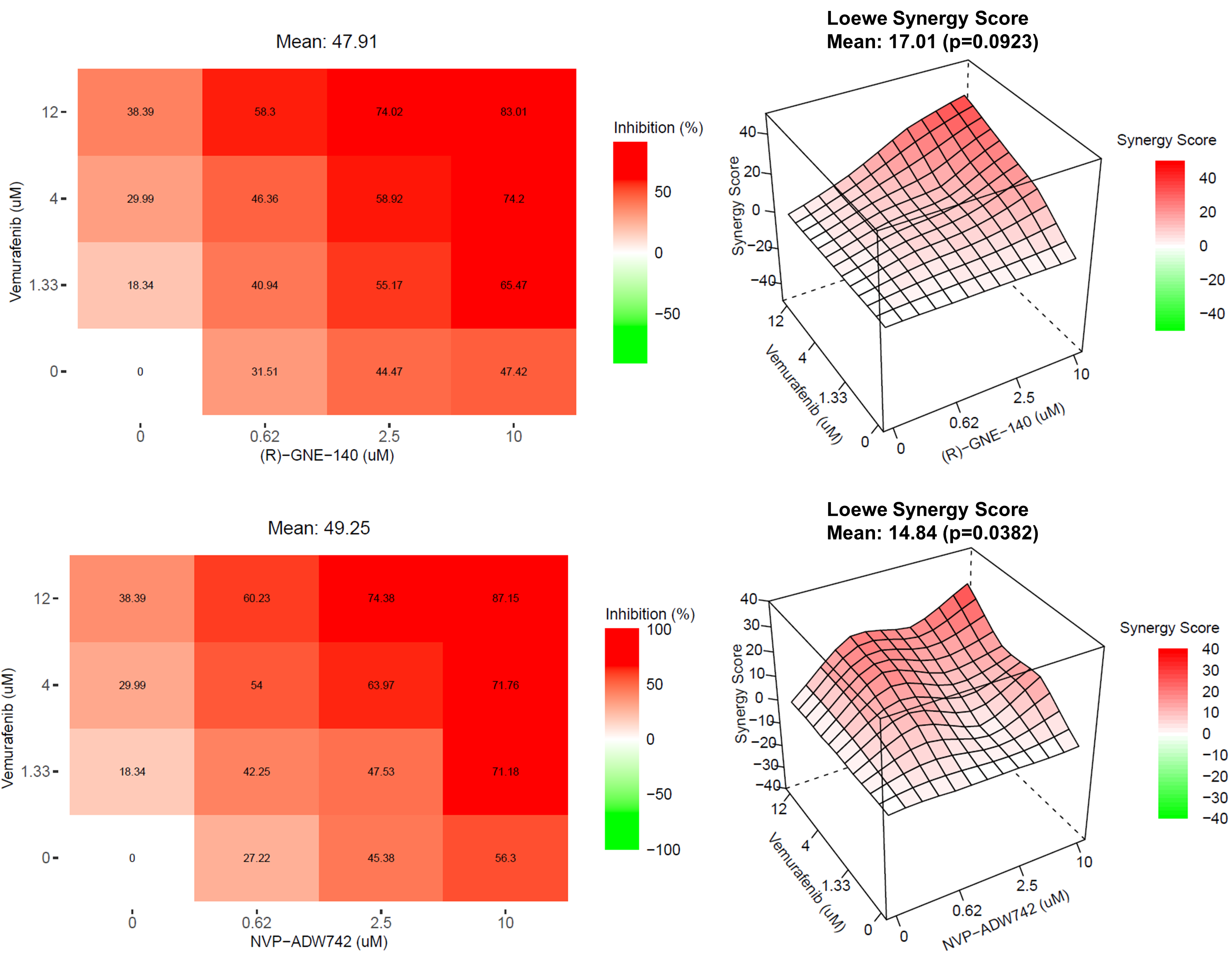
**

**Fig. S10 Drug synergy data for Hs294T cells**
